# Comparison of medical students' considerations in choosing a specialty: 2020 vs. 2009/10

**DOI:** 10.1186/s12960-023-00885-7

**Published:** 2024-01-08

**Authors:** Hanna Schroeder, Alon Shacham, Shimon Amar, Charles Weissman, Josh E. Schroeder

**Affiliations:** 1 Policy Planning Division at the Israel Ministry of Health, Jerusalem, Israel; 2 Henrietta Szold School of Nursing, Hebrew University - Hadassah Faculty of Medicine, Jerusalem, Israel; 3 Hebrew University Faculty of Medicine, Jerusalem, Israel; 4 Shaare Zedek Medical Center, Jerusalem, Israel; 5 Hadassah Medical Center, Hebrew University of Jerusalem, Jerusalem, Israel; 6 Joyce and Irving Goldman School of Medicine and Department of Family Medicine, Faculty of Health Sciences, Ben-Gurion University of the Negev, Be’er Sheva, Israel

**Keywords:** Medical students, Medical specialties, Generation Y, Residency, Career

## Abstract

**Background:**

Workforce shortage in healthcare and particularly in physicians poses a threat to healthcare delivery and its quality. In comparison to other OECD countries, Israel currently has a small number of medical graduates relative to its number of physicians, naturally emphasizing the importance of ensuring that this population chooses to remain in medicine. Understanding what is most important to medical students can help improve working conditions in residency. Such information is particularly needed to facilitate policy planning that will encourage the next generation of physicians to specialize in medical fields that are experiencing shortages. We hypothesized that between 2009/2010 and 2020, there were significant changes in medical students' preferences regarding their considerations for choosing a medical specialty.

**Methods:**

We compared cross-sectional data from questionnaire-based surveys of 5th year medical students performed in 2009–2010 and 2020 at two Israeli universities.

**Results:**

Of the 335 medical students who responded (237 and 98 in 2009/2010 and 2020, respectively) those in 2020 were 2.26 less likely vs. those in 2009/2010, to choose a residency for its high-paying potential (*P* < 0.05), and had significantly more interest in residencies with greater teaching opportunity (98.8% vs 82.9%, *P* < 0.05), increased responsibility and chances to make clinical decisions on their own (67.9% vs 51.6%, *P* < 0.05). Criteria important to both the 2009/2010 and 2020 students were choosing a bedside specialty (70.2%vs 67.9%, NS), and an interesting and challenging specialty (95.2%v s 91.3%, NS).

**Conclusions:**

These results partially supported our hypothesis that medical students' preferences have changed over the years, though there are fundamental factors that apparently reflect medical students’ nature that do not change over time.

## Introduction

An important element of quality healthcare is ensuring that there are sufficient numbers of specialist physicians in all the medical fields. Planning for the anticipated demand for professional personnel is a strategic objective of healthcare-systems worldwide [1]. This is a major concern in Israel which has 10% fewer physicians per 1000 population than the OECD average. The number of physicians per capita is projected to continue to decline over the next 10 years. According to a recent OECD report, Israel lacks a long-term plan for residency and specialty training. This lack of planning has resulted in fluctuations in the number of specialists and has failed to meet the needs of the population [2]. In response, the Ministry of Health has created a new division dedicated to healthcare workforce planning [2].

As of 2021, there were 6 medical schools in Israel with a total of 907 beginning medical students. Most of the students studied at Tel Aviv University (22%) and The Hebrew University of Jerusalem (21%), following by Ben Gurion University in Be’er Sheva (18%) and the Technion University in Haifa (15%). The number of medical students in Israeli medical schools has grown gradually by 170% over the past decade. However, in recent years the majority of Israeli born doctors have obtained their medical degree abroad [2].

Understanding what is most important to medical students can help improve specialty and residency selection, as well personal satisfaction. Avoiding stereotypes that are based on the generational cohort and instead asking medical students directly what are their needs and motivations, has been stressed by others [3]. This method can facilitate short- and long-term policies that will encourage the next generation of physicians to specialize in medical specialties expected to experience workforce shortages [4].

Previous studies from a variety of countries explored the criteria medical students use when selecting a career specialty. These studies found that influencers (clerkship experiences, mentors, junior and senior physicians), lifestyle (work–life balance, control over life style), personal attributes (personality, interests, gender, marital status), socioeconomic considerations (future earnings, loan repayments) and perceptions of the various specialties are important criteria when choosing a specialty and a residency program [5–8]. The leading criteria in many studies were the ability to have a controllable lifestyle and finding the specialty interesting and challenging. These leading criteria were also found among Israeli medical students [12].

The recent decline in medical students choosing specialties that require long working hours and have unpredictable work schedules such as general surgery [9, 10], has been attributed to a generational shift [10]. Millennials (Generation Y), described as persons born between 1980 and 1994 [11, 12], are more inclined than previous generations to emphasize the importance of work–life balance [13] and garnering personal appreciation [14]. However, few studies have compared changes in the choice of medical specialty selection over time [15], and none have been performed in Israel. The aim of this study was to compare the importance of certain criteria used by medical students when choosing a medical specialty in 2020 vs. data collected in 2009/10 and subsequently published [16]. We hypothesized that there would be significant changes in student preferences over time.

## Methods

### Study design

This cross-sectional study was prepared according to the Strengthening the Reporting of Observational studies in Epidemiology (STROBE) statement guidelines for cross-sectional studies [17].

### Settings and participants

In 2009/10 and in 2020, online questionnaires were distributed via the university deans' offices directly by e-mail to the 5th year medical students from two Israeli medical schools: The Hebrew University-Hadassah School of Medicine in Jerusalem and the Joyce and Irving Goldman School of Medicine of Ben-Gurion University in Beer-Sheva.

### Questionnaires

The questionnaire used in the 2009/10 study, designed to examine various aspects of the specialty and residency selection processes [16], was validated with two pilot studies. We used the same questionnaire in 2020. The questions were multiple choice and responses were on 4- and 5-point Likert scales. In addition to demographic information, the questionnaire elicited information on (1) reasons for choosing a medical career, (2) criteria for choosing a career specialty and (3) criteria for choosing a residency program.

### Statistical methods

The responses to multiple choice questions are presented as frequency distributions. Analysis of the replies to the 4-point Likert scales involved combining the two positive and two negative points; while analysis of the 5-point Likert scale involved combining the two positive and two negative points. Principal Component Factor Analysis (PCA) following listwise deletion and using oblique rotation examined questionnaire construct validity.

We used descriptive statistics, Pearson chi-square, and Fisher-exact tests to compare 2009/10 with 2020 data. Cramer's-V served as a measure for effect size. We used logistic regression to explore the association of choosing a high-income specialty (dependent variable) with criteria for choosing a specialty and residency program and demographic variables (independent variables), while controlling for age and sex.

The data were analyzed using IBM SPSS Statistics for Windows Version 27 (IBM Corp., Armonk, NY).

### Ethics approval

The Institutional Review Board of the Hadassah Medical Organization approved this study. Completion of the questionnaire by the students was considered tacit consent.

## Results

Of 335 5th year medical students who answered the survey, 237 (54% of the students) did so in 2009/10 and 98 (37% of the students) in 2020. The demographic characteristics (sex and age) of the respondents were similar to the total population of medical students. There were no significant differences in the characteristics of the medical student respondents in 2020 vs those in 2009/10, except for age (Table 1)**.**

**Table 1:** Summary of the participating students

	2009/10 Medical students	2020 Medical students
Female, *N*, (%)	105 (44.3)	51 (52)
Single, *N*, (%)	151 (63.7)	61 (62.3)
Age-group, N, (%)		
21–23 years	44 (18.6)	25 (26)
24–26 years	65 (27.4)	21 (21.4)
27–29 years	90 (38)	29 (30)
30–32 years	38 (16)*	23 (23.5)*

*2020 vs 2009/10: *P* value < 0.05

The 2020 group more often reported their preference for a medical career with economic potential, scientific basis and employment security, than did the 2009/10 group (Table 2).

**Table 2:** Reasons for choosing a medical career

Question (Factor)	All (335)	2010 (237)	2020 (98)
Helping patients (3)	94.4%	92.7%	98.8%
The scientific basis of medicine (3)	76.2%	71.2%	89.3%*
Employment security (1)	67.8%	59.1%	90.5%‡
The profession’s image (4)	63.2%	62.7%	64.3%
Economic potential (1)	47.7%	40.5%	66.6%*
Influence of a role model (family/friends) (4)	31%	30.1%	30.1%
Influence of a role model (physician) (4)	34.8%	33.9%	36.9%
Opportunity to perform research (3)	38.6%	36.8%	43.4%
Potential for family life (1)	27.7%	25.1%	34.5%
Illness of relative/friend (2)	27%	25.5%	31%
Previous employment experience (2)	10.3%	10.7%	9.5%

Values are the important/very important answers on a 4-point Likert Scale

2009/10 vs. 2020: *P < 0.05, ^‡^*P* < 0.01

Factors are the result of the factor analysis performed on all the data

**Fig. 1:**
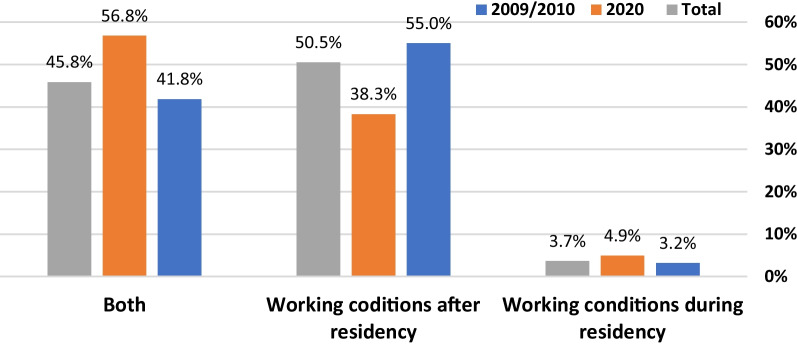
Responses to the query: which is more important when choosing a specialty, working conditions after residency, working conditions during residency, or both?

**Fig. 2:**
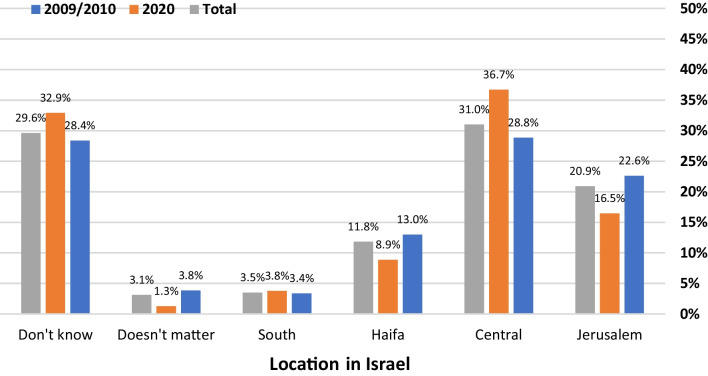
The locations where the medical students would like to do their residency training. The Hebrew University—Hadassah School of Medicine is located in Jerusalem, while the Ben-Gurion University Joyce and Irving Goldman School of Medicine is in the Southern Region. The Central Region is the major population center (Tel Aviv and its environs)

The 2020 respondents were significantly more interested than their 2009/2010 colleagues in residencies that included more teaching, increased clinical responsibility, and a greater chance to make clinical decisions on their own. There were criteria which were important to both the 2010 and 2020 students, such as, choosing a bedside specialty  and an interesting and challenging residency (Tables 3 & 4). The 2009/10 students cared more about working conditions after residency (55% in 2009/10 vs 38.3% in 2020, *P* < 0.05) while 2020 students emphasized conditions both during and after residency (Fig. 1). The importance of a residency's geographic location was considered more important for 2020 students than 2009/10 students (Table 4). The 2020 data showed that the popularity of a residency in the country's center continued to increase (28.8% in 2009/2010 vs 36.7% in 2020) as opposed to a residency in Haifa and the northern periphery (13% in 2009/2010 vs 8.9% in 2020) and continued avoidance of a residency in the southern periphery (3.4% in 2009/2010 vs 3.8% in 2020) (Fig. 2).

**Table 3:** Which of the following will have a positive influence on your selection of a specialty as a career?

Question (Factor)	All (335)	2009/10 (237)	2020 (98)
Interesting/challenging specialty (1)	92.4%	91.3%	95.2%
A reasonable relationship between salary/lifestyle (2)	73.6%	72.6%	76.2%
Control over lifestyle (2)	69.5%	67.0%	76.2%
Bedside specialty (1)	68.5%	67.9%	70.2%
A specialty that is rapidly advancing (1)	58.3%	56.9%	61.9%
Independent practice (2)	52.6%	54.1%	48.8%
Opportunity for private practice (2)	47.5%	47.5%	47.6%
High-paying specialty (2)	47%	49.5%	40.5%
Performing surgery/procedures (3)	45.9%	46.1%	45.2%
Much teamwork (1)	39.4%	36.7%	46.4%
Time in the operating room (3)	31.8%	32.6%	29.8%
Work only during the daytime (2)	30.8%	28.4%	36.9%
Opportunity to perform research (1)	29.8%	29.8%	29.8%
Work only in the hospital (2)	13.3%	13.8%	11.9%
Specialties classmates choose	1.3%	0.5%	3.6%*

Values are important/very important answers on a 5-point Likert Scale

2009/10 vs. 2020: *P < 0.05

Factors are the result of the factor analysis performed on all the data

**Table 4:** Which of the following will have a positive influence on your selection of a residency program?

Question (Factor)	All (335)	2009/10 (237)	2020 (98)
Good relationships with attendings (3)	92.6%	91.2%	96.3%
Good relationships between residents (3)	91%	89%	96.3%
Interesting and challenging residency (4)	87.7%	86.8%	90.1%
Residency with much resident teaching (3)	87.2%	82.9%	98.8%*
Leading department in the specialty (2)	73.3%	72.6%	75.3%
A specific location within Israel	66.7%	60.7%	82.7%*
Control over lifestyle (1)	65%	63.9%	67.9%
Much responsibility/make clinical decisions on their own (4)	56%	51.6%	67.9%*
A large university hospital (2)	54%	57.1%	45.7%
Working hours known from the start (1)	51.8%	49.1%	59.3%
A residency with much “action" (4)	42.3%	42.9%	40.7%
Residency with limited hours (1)	33%	31.5%	37%
A residency with few on-calls	30.7%	29.2%	34.6%
Opportunity to perform research (2)	24.7%	23.3%	28.4%
Work under pressure (4)	19.4%	17.4%	24.7%*
Short residency (< 4.5 years) (1)	19.1%	20.6%	14.8%
Hospital in the country’s periphery (2)	6.7%	4.6%	12.3%*

Values are important/very important answers on a 5-point Likert Scale. *P < 0.05. Factors are the result of the factor analysis performed on all the data

Logistic regression revealed that students in 2009/10 were more likely than 2020 students (OR = 2.16, CI 95% 1.2–4.26, *P* < 0.05) to consider a residency in a "*high paying specialty*". Interest in a high-paying specialty was also associated with a specialty (OR = 2.52, CI 95% 0.26–0.79 *P* < 0.05) and a residency (OR = 3.52, CI 95% 0.16–1.14, *P* < 0.05) that enables control over lifestyle.

### Location of residency

Logistic regression revealed that the importance of the selection criteria varied depending on respondents’ preference of their residency's location. Students interested in a residency in the country's center also expressed more interest in a residency that enables control over their lifestyle (OR = 4.02, CI 95% 1.01–15.07, *P* < 0.05) and that has a potential for high income (OR = 3.1, CI 95% 1.3–7.45, *P* < 0.05). Students interested in a residency in Jerusalem were older (OR = 3.78, CI 95% 1.2–11.6, *P* < 0.05) and less interested in specialty that involves teamwork (OR = -4.3, CI 95% 0.094–0.6, *P* < 0.05).

## Discussion

This study provided insights on what is most important to medical students when they are asked to choose a specialty for residency, as well as a residency program. The results demonstrated that medical students in 2020 were more interested than their predecessors in residencies with much resident teaching as well as the chance and responsibility to make clinical decisions on their own. The latter likely reflects this generation's overlap with Generation Z (born between 1995 and 2010 [18]) which is characterized by its independence [19], along with their own Generation Y's result-oriented character [4]. However, the present study also found no differences between the 2009/10 group vs. the 2020 group in the importance of some criteria for selecting a medical specialty or residency. This trend is not unexpected and reinforces the idea that certain considerations are universal over time: the importance of an interesting and challenging career specialty; a specialty with considerable bedside interaction and a specialty and residency that enable control over one’s lifestyle were important to 2020 respondents as well as in studies performed over a decade earlier (2000–2009) [20–24].

Our results demonstrated that there are fundamental factors of human nature, and in this case medical students’ nature, that change little or slowly over time. Therefore, recruitment initiatives, aimed at attracting medical students to a specific specialty that is typically less popular, should demonstrate how the specialty meets the baseline criteria of being interesting and challenging, enabling a reasonable relationship between work and lifestyle, and offering direct patient contact as well as the opportunity to teach, take responsibility and make decisions.

In both 2009/10 and 2020, students emphasized the importance of choosing a residency where there are good relationships with attendings and other residents. These criteria reflect the basic human need to be supported and respected [25–27]. A study performed among anesthesiology residents in Germany stressed the need for a positive and respectful working climate that contributes to good training conditions [28]. Millennials have been found to emphasize the need for achievement and affiliation [24]. This finding is especially relevant to the flat social structure and concept of hierarchical structures possessed by Millennials and the overlapping Generation Z, that differentiate them from previous generations [29]. They have grown up in the internet-age and are accustomed to communicating openly with senior figures on social media; they are less intimidated by direct contact with their attendings [30] and function more willingly as part of a multidisciplinary team [24, 31, 32]. This was demonstrated in the present study where the 2020 group rated a specialty that emphasized teamwork more highly than did 2009/10 students.

Previous studies have also reported that today’s medical students place more emphasis on a specialty with flexible working conditions than a higher salary.[4, 33, 34] Two time-comparison studies of career preferences found that medical students and residents are more likely (*P* < 0.05) than their predecessors to emphasize the importance of a career that enables work–life balance, and that has stable working hours [9, 15]. Members of Generation-Y are committed to working hard and also expect to take an active role at home and express uncertainty regarding how future plans will influence them holistically [35]. These shifts may also be explained partially by the fact that the majority of medical students in 2020 are female [36] who, in comparison to males, have different expectations from their medical career [37, 38]. Female medical students have also been reported [20] to emphasize the importance of lifestyle when choosing a specialty and the desire to work fewer hours than male physicians [39]. Thus, medical students may avoid choosing specialties such as general surgery that are in need of a greater number of residents but provide less control over lifestyle [20, 40, 41] as opposed to family medicine, which offers a more controllable lifestyles [38]. This situation will likely not change since high-school students belonging to Generation Z also aim for a good work–life balance [42].

The 2020 group almost unanimously replied that they were interested in choosing a residency program that can offer them didactic teaching. This desire for an ongoing connection with learning likely reflects Millennials' and Generation Z's interest in frequently updating their skills and knowledge to advance in their careers. Notably, they are also accustomed to assimilating information quickly, especially from digital sources, which means adapting teaching techniques to the use of technology in learning [13, 43, 44]. Suggested guidelines for the teaching of millennials by non-millennials [45, 46] have indicated that educators need to give detailed instructions [47], supply prompt feedback [31, 48] and provide for micro-learning [49] and micro-mentoring [30]. The current study provides information that can shape the learning environment of medical undergraduate and graduate education programs.

## Choosing medicine as a career

Medical students in 2020, when compared to those in 2009/10, emphasized the importance of choosing a medical career with employment and financial security. This greater emphasis on economic security could possibly be explained by their having grown up during the 2008 recession, when unemployment rates were high (2009—9.5%, 2009/10—8.5%) [50]. At the same time, the 2020 group associated less importance to a specialty with high pay when compared to the 2009/10 group. This phenomenon was also described previously in a study of first-year medical students [9]. Perhaps into medical school and the medical profession in general represents employment security, and they are then more open to choosing a specialty that allows for life–work balance. Another possible explanation is that in 2020 the questionnaires were distributed during the COVID-19 pandemic, when medical professionals enjoyed employment security unlike many other workers.

## Implications for policy health planning and medical education

The findings of this study can help health policy planners and medical educators assist medical students in their choice of a career medical specialty and residency program. Department chairs and residency program directors can improve residency learning and working conditions to make their specialty more attractive. Adjusting residency to include more teaching, enabling residents to make more autonomous clinical decisions, and modifying work hours to allow for a better work–life balance, may increase the willingness of today’s (and tomorrow’s) students to select specialties in which there are workforce shortages. Better structured training programs, which foster a friendly and respectful environment starting with medical school rotations and through residency, may also attract new residents. Medical school administrators need to query the students at regular intervals to determine whether new selection criteria have become important. Other studies [32] have recommended exploring the needs and motivations of medical students and avoiding stereotypes that are based on the generational cohort.

Although Israel is a relatively small country, its northern and southern peripheral areas suffer from a shortage of physicians and certain specialists [51, 52]. The present study demonstrated that like their counterparts 10 years previously, the 2020 group continued to be very interested in residencies in the country's center and only a very small minority were interested in residencies in a peripherally located hospital. Importantly, wanting to do their residency in the country's center was associated with the selection criteria "*a high paying specialty*" and "*control over lifestyle*". This reflects Israel's intrinsic and major dilemma, namely that the population, employment opportunities and healthcare infrastructure and personnel are increasingly concentrated in the small central geographic area, while the peripheral areas, although not especially distant geographically, are lacking personnel and infrastructure [52, 53]. Monetary incentives, one-time grants and higher salaries, incorporated into the 2011 physician's union contract to attract residents to peripheral hospitals were only partially successful [54]. Perhaps policy measures, in addition to purely monetary ones, should also be considered. As the present study shows, these non-monetary incentives could include improving control over lifestyle, more flexible work hours and possibly enabling the opening of private practices. A qualitative study reports that residents are inclined to stay in the periphery if they are promised a specialist position with good conditions [53].

## Strengths and limitations

The strength of this study is that it is among the first to re-examine specialty and residency program selection criteria using the same questionnaire in order to learn whether there have been any changes over time.

A possible limitation is that the 2020 group had a larger proportion of students in the young and older age groups. Yet, despite the age distribution differences between the two groups, the importance of most of the selection criteria did not change. This study participants were medical students from two schools; other studies [55] have reported that a student's medical school may also influence their choice of specialty, therefore we suggest that future studies explore the preferences of students in the other Israeli medical schools.

Another limitation is that both medical schools underwent curricular changes between the survey dates as well as changes in the demographics of their student bodies. For example, in 2020, The Hebrew University Hadassah School of Medicine included students in a pre-military program who were admitted immediately after high school graduation, thus lowering the class age. Moreover, the residency choice of these students is significantly delayed by their post-medical school military commitment. In a previous paper, it was noted that despite the pre-military subgroup being younger and having another 7 years of medical school, internship and military service before residency, they had begun thinking about which specialty to choose, just like their older colleagues [56]. In addition, admission criteria to the Hebrew University changed between the surveys to emphasize non-cognitive attributes which, according to one paper, led to career choice differences [9]. Yet, despite the changes in many of the selection criteria, there were no or minimal differences between the two time periods of our study, once again demonstrating that many criteria are universally important over time. Finally, the study was performed during the COVID-19 pandemic, which may have influenced medical students to rethink their professional choices in term of work–life balance and burnout [57].

## Conclusions

"The more things change, the more they stay the same” (1849, French writer Jean-Baptiste Alphonse Karr), i.e., many things remain constant, even as changes are occurring. Over the past decade, the importance of some specialty and residency program selection criteria did not change while others did. The former criteria should be considered by the healthcare leadership, medical educators, clinical department chairs and residency program directors as basic issues to be highly emphasized when attempting to attract medical students to the various specialties and residency programs, especially specialties with workforce shortages. Without taking advantage of this information, strategic and tactical planning for the specialty composition of the future physician workforce will be hindered by a lack of knowledge of the students' considerations and concerns. In many countries, including Israel these issues, especially the emphasis placed by students on life–work balance, are important for the healthcare and medical education leadership to internalize given the impending overall shortage of physicians, the imbalanced geographic distribution and the uneven distribution among the various specialties.

## Data Availability

The datasets used and/or analyzed during the current study are available from the corresponding author on reasonable request.
